# Characterization of Nyquist ghost in EPI‐fMRI acquisition sequences implemented on two clinical 1.5 T MR scanner systems: effect of readout bandwidth and echo spacing

**DOI:** 10.1120/jacmp.v11i4.3237

**Published:** 2010-07-12

**Authors:** Marco Giannelli, Stefano Diciotti, Carlo Tessa, Mario Mascalchi

**Affiliations:** ^1^ Unit of Medical Physics Azienda Ospedaliero‐Universitaria Pisana Pisa Italy; ^2^ Radiodiagnostic Section, Department of Clinical Physiopathology University of Florence Firenze Italy; ^3^ Biomedical Engineering Laboratory, Department of Electronics and Telecommunications University of Florence Firenze Italy; ^4^ Unit of Radiology Versilia Hospital Lido di Camaiore (LU) Italy

**Keywords:** magnetic resonance imaging, fMRI, echo planar imaging, echo spacing, readout bandwidth, Nyquist ghost

## Abstract

In EPI‐fMRI acquisitions, various readout bandwidth (BW) values are used as a function of gradients' characteristics of the MR scanner system. Echo spacing (ES) is another fundamental parameter of EPI‐fMRI sequences, but the employed ES value is not usually reported in fMRI studies. Nyquist ghost is a typical EPI artifact that can degrade the overall quality of fMRI time series. In this work, the authors assessed the basic effect of BW and ES for two clinical 1.5 T MR scanner systems (scanner‐A, scanner‐B) on Nyquist ghost of gradient‐echo EPI‐fMRI sequences. BW range was: scanner‐A, 1953‐3906 Hz/pixel; scanner‐B, 1220‐2894 Hz/pixel. ES range was: scanner‐A, scanner‐B: 0.75‐1.33 ms. The ghost‐to‐signal ratio of time series acquisition $\left({\text{GSR}}_{\text{ts}}\right)$ and drift of ghost‐to‐signal ratio $\left({\text{DR}}_{\text{GSR}}\right)$ were measured in a water phantom. For both scanner‐A (93% of variation) and scanner‐B (102% of variation) the mean ${\text{GSR}}_{\text{ts}}$ significantly increased with increasing BW. ${\text{GSR}}_{\text{ts}}$ values of scanner‐A did not significantly depended on ES. On the other hand, ${\text{GSR}}_{\text{ts}}$ values of scanner‐B significantly varied with ES, showing a downward trend (81% of variation) with increasing ES. In addition, a ${\text{GSR}}_{\text{ts}}$ spike point at ES=1.05ms indicating a potential resonant effect was revealed. For both scanners, no significant effect of ES on ${\text{DR}}_{\text{GSR}}$ was revealed. ${\text{DR}}_{\text{GSR}}$ values of scanner‐B did not significantly vary with BW, whereas ${\text{DR}}_{\text{GSR}}$ values of scanner‐A significantly depended on BW showing an upward trend from negative to positive values with increasing BW. ${\text{GSR}}_{\text{ts}}$ and ${\text{DR}}_{\text{GSR}}$ can significantly vary with BW and ES, and the specific pattern of variation may depend on gradients performances, EPI sequence calibrations and functional design of radiofrequency coil. Thus, each MR scanner system should be separately characterized. In general, the employment of low BW values seems to reduce the intensity and temporal variation of Nyquist ghost in EPI‐fMRI time series. On the other hand, the use of minimum ES value might not be entirely advantageous when the MR scanner is characterized by gradients with low performances and suboptimal EPI sequence calibration.

PACS numbers: 87.61.‐c, 87.61.Qr, 87.61.Hk

## I. INTRODUCTION

Functional magnetic resonance imaging (fMRI) based on blood‐oxygen‐level‐dependent (BOLD) contrast^(^ ^1^ ^)^ has advanced the field of brain research by noninvasively enabling imaging of brain function with a spatial and temporal resolution on the order of millimetres and seconds, respectively. fMRI techniques^(^ ^2^ ^)^ have been widely used to detect human brain activity changes associated with motor, sensory or cognitive processes.^(^ ^3^ ^)^

The BOLD response is an indirect measure of neural activity. The relationship between BOLD contrast and cerebral oxygen metabolism can be influenced by a number of physiological factors.^(^ ^4^ ^,^ ^5^ ^)^ Nonetheless, in fMRI the signal changes associated with BOLD contrast depend also on static magnetic field strength, hardware characteristics of the MR scanner system, radiofrequency (RF) coils configuration, in addition to acquisition parameters in terms of acquisition sequence, repetition time, echo time and voxel size.^(^ ^6^ ^,^ ^7^ ^)^ Logothetis^(^ ^5^ ^)^ has recently analyzed the potential limits of fMRI applications from a theoretical analysis of physiological processes involved in BOLD effect. Previous studies have assessed in vivo the reliability of fMRI analyses and possible practical limitations.^(^ ^8^ ^–^ ^14^ ^)^ Moreover, the reproducibility and variability of fMRI studies have been investigated as well.^(^ ^15^ ^–^ ^19^ ^)^ Vlieger et al.,^(^ ^20^ ^)^ Costafreda et al.,^(^ ^21^ ^)^ Friedman et al.^(^ ^22^ ^)^ and Bosnell et al.^(^ ^23^ ^)^ have studied multisite fMRI reproducibility. Fera et al.^(^ ^24^ ^)^ have evaluated in vivo the sensitivity dependence of activation clusters on acquisition bandwidth and echo time. Zou et al.^(^ ^25^ ^)^ have reported the effects of readout bandwidth on measured activation maps in clinical fMRI experiments. Some studies have performed comparison analyses of the effect of processing parameters of fMRI time series on activation maps.^(^ ^26^ ^–^ ^28^ ^)^

**Figure 1:**
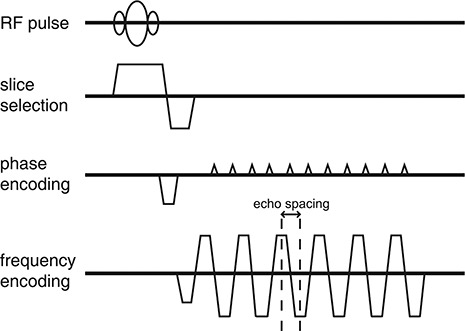
Graphical diagram of gradient‐echo EPI‐fMRI acquisition sequence. The echo spacing represents the interval between successive echoes.

In fMRI, the signal change due to BOLD contrast is typically less than 5% at 1.5 T and not much higher than the general noise components in acquisition time series. An MR scanner system should allow the acquisition of fMRI images with sufficient signal‐to‐noise ratio for accurately revealing such a small signal change.^(^ ^29^ ^)^ In particular, the intrinsic fluctuation levels of fMRI time series signal and any temporal signal drift should be lower than the expected BOLD signal change. Thus, the hardware characteristics and sequence parameters should be optimized to obtain the best performances of MR scanner in acquisition of fMRI data. In this regard, readout bandwidth (BW) and echo spacing (ES) are fundamental parameters in echo planar imaging (EPI)‐fMRI sequences. BW is related to image field of view along frequency encoding direction $\left({\text{FOV}}_{r}\right)$ and readout gradient strength $\left(G_{r}\right)$ as it follows:^(^ ^30^ ^)^

(1) $$BW=\frac{\gamma }{2\pi }\cdot G_{r}\cdot FOV_{r}$$

where γ is the gyromagnetic ratio of hydrogen proton (γ/2π=42.58MHz/T). On the other hand, ES represents the interval between successive echoes in EPI acquisition sequence (Fig. 1).

EPI techniques have some drawbacks such as Nyquist or N/2 ghost and geometric distortion in the reconstructed images. Thus, additional calibration methods and postprocessing are required to reduce these artifacts.^(^ ^31^ ^–^ ^38^ ^)^ Nyquist ghost is a typical artifact of EPI‐fMRI images which arises from phase difference due to any asymmetry between the odd and even echoes that constitute an EPI dataset. This results in a ghost image which is shifted by half the field of view in the phase encode direction. Nyquist ghost is expected to have a deleterious effect on EPI‐fMRI examinations: it can mask BOLD signal change due to functional activation, or it can add an undesirable signal drift to EPI‐fMRI time series. To our knowledge, so far no study has quantitatively assessed the effect of both BW and ES on intensity and temporal variation of Nyquist ghost of EPI‐fMRI time series. In the present work we characterized, by means of phantom measurements, the basic effect of BW and ES on Nyquist ghost of EPI‐fMRI acquisition sequences implemented on two clinical 1.5 T MR scanners with different gradients and radiofrequency systems.

## II. MATERIALS AND METHODS

### A. MR scanners and phantom

All fMRI acquisitions were performed on two commercial 1.5 T MR scanner systems: Signa HDx TwinSpeed (GE Medical Systems, Milwaukee, WI, USA) with 50mT/m maximum gradient strength and 150T/m/s slew rate (scanner‐A) and MAGNETOM Symphony (Siemens, Erlangen, Germany) with 30mT/m maximum gradient strength and 75mT/m/s slew rate (scanner‐B). Scanner‐A was equipped with a standard quadrature head coil for RF transmission and reception of the NMR signal. Scanner‐B was equipped with a standard quadrature head coil which only receives the NMR signal while the RF transmission is carried out by the body coil. For all data acquisitions the same cylindrical water phantom (diameter 16 cm, length 36 cm) was employed.

### B. Data acquisition

Images from scanner‐A and scanner‐B were obtained using vendor‐provided pulse sequence and reconstruction software. For data acquisition of each MR scanner a standard gradient‐echo EPI sequence was used. The acquisition parameters were those employed in a typical fMRI study: TR 3000 ms, TE 50 ms, field of view 24 24×24 cm, matrix 64×64, slice thickness 5 mm, interslice gap 1 mm, number of slices 21, number of excitation 1. Eighty volumes/scan were acquired.

fMRI acquisitions were obtained with independently varying readout bandwidth and echo spacing at fixed ES and BW, respectively. The used combinations of BW/ES values for scanner‐A and scanner‐B are reported in Table 1. For each MR scanner system, all acquisitions were consecutively performed on the same day avoiding any long‐term changes of the scanner performances and any potential variability in phantom repositioning. The order of fMRI acquisitions with different BW/ES values was randomized to avoid any bias in the data. The entire set of acquisitions with different BW/ES values was repeated three times with different randomized orders, resulting in a total of three measurements for each BW/ES combination.

**Table 1:** The BW and ES values employed for fMRI acquisitions of scanner‐A and scanner‐B.

	*Scanner‐A*	
*BW values* (Hz/pixel) *at fixed* ES=0.9ms		*ES values (ms) at fixed* BW=1953Hz/pixel
1953		0.75
2604		0.90
2841		1.05
3125		1.20
3472		1.33
3906		
	*Scanner‐B*	
*BW values* (Hz/pixel) *at fixed* ES=0.9ms		*ES values (ms) at fixed* BW=1502Hz/pixel
1220		0.75
1370		0.90
1502		1.05
1906		1.20
2368		1.33
2894		

**Figure 2:**
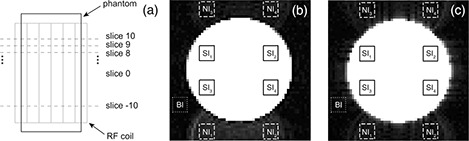
Set up of water phantom acquisitions (a); measurements of ${\text{GSR}}_{\text{ts}}$ and ${\text{DR}}_{\text{GSR}}$ were performed in slice 0 which is the median slice of the acquired phantom volume. Scanner‐A (b): images with BW=1953Hz/pixel and ES=0.9ms. Scanner‐B (c): images with BW=1906Hz/pixel and ES=0.9ms. The ROIs (b, c) used for the measurement of signal intensity within the phantom $\left({\text{SI}}_{i},\;i=1,\;2,\;3,\;4\right)$ (solid line), and the ROIs used for the measurement of signal intensity of Nyquist ghost $\left({\text{NI}}_{i},\;i=1,\;2,\;3,\;4\right)$ (dashed line) and background (BI) (dotted line), are shown. The ROIs employed for the measurement of ${\text{NI}}_{i}\;(i=1,\;2,\;3,\;4)$ are shifted by N/2 voxels (64/2) along the phase encoding direction in respect to the corresponding ROIs employed for the measurement of ${\text{SI}}_{i}\;(i=1,\;2,\;3,\;4)$.

The center of the water phantom was placed in the center of the head coil by means of foam padding. Moreover, the center of the acquisition slab was placed in the center of the water phantom (Fig. 2(a).

### C. Image processing and analysis

Processing of fMRI time series was performed by using custom scripts software in MATLAB 7.0 (MathWorks, Natick, MA, USA) running on a personal computer. The effect of different BW and ES values on Nyquist ghost of fMRI acquisitions was investigated measuring two parameters. The intensity of Nyquist ghost was characterized by the overall ghost‐to‐signal ratio of fMRI time series acquisition $\left({\text{GSR}}_{\text{ts}}\right)$. The temporal variation of Nyquist ghost was assessed by the drift of ghost‐to‐signal ratio $\left({\text{DR}}_{\text{GSR}}\right)$. The measurements were performed in the median slice (slice 0) of the acquired phantom volume (Fig. 2(a).

In order to calculate ${\text{GSR}}_{\text{ts}}$, four symmetrical ROIs (7×7voxels) were placed in phantom regions (Fig. 2(b), 2(c)) and their mean signal intensity was measured (${\text{SI}}_{i},\;i=1,\;2,\;3,\;4)$. For each ROI, the mean signal intensity (${\text{NI}}_{i},\;i=1,\;2,\;3,\;4)$ of a corresponding ROI shifted by N/2 voxels (64/2) along the phase encoding direction was obtained (Fig. 2(b), 2(c)). The mean background intensity (BI) was measured within a 7×7voxels ROI placed in the ghost‐free background (Fig. 2(b), 2(c)). For the volume j–th, the ghost‐to‐signal ratio (${\text{GSR}}_{j}$) was calculated as:

(2) $$GSR_{j}=\frac{1}{4}\sum_{i=1}^{4}\frac{{\left(NI_{i}\right)}_{j}‐{\left(BI\right)}_{j}}{{\left(SI_{i}\right)}_{j}}$$

Then, ${\text{GSR}}_{\text{ts}}$ was computed averaging the time series of recorded ghost‐to‐signal ratio values across the 80 volumes:

(3) $$GSR_{ts}=<GSR_{j}>=\frac{1}{80}\sum_{j=1}^{80}GSR_{j}$$

As for calculation of ${\text{DR}}_{\text{GSR}}$, a second order polynomial trend was employed to fit the time series of ghost‐to‐signal ratio values (${\text{GSR}}_{j}$). The ${\text{DR}}_{\text{GSR}}$ was computed by subtracting the minimum fit value ($\text{Min}({\text{GSR}}_{\text{fv}})$) from the maximum fit value ($\max({\text{GSR}}_{\text{fv}})$) and dividing by the mean value ($\text{Mean}({\text{GSR}}_{\text{fv}})$). Finally, the ${\text{DR}}_{\text{GSR}}$ value was multiplied by 100 times the sign of difference between final fit value $\left({\text{GSR}}^{80‐\text{fv}}\right)$ and initial fit value $\left({\text{GSR}}_{1‐\text{fv}}\right)$, obtaining a percentage of variation:

(4) $$DR_{GSR}=100\frac{Max(GSR_{fv})‐Min(GSR_{fv})}{Mean(GSR_{fv})}sign(GSR_{80‐fv}‐GSR_{1‐fv})$$

Positive ${\text{DR}}_{\text{GSR}}$ values indicate a main upward drift of ghost‐to‐signal ratio, whereas negative ${\text{DR}}_{\text{GSR}}$ values indicate a main downward drift of ghost‐to‐signal ratio.

Any significant difference of ${\text{GSR}}_{\text{ts}}$ and ${\text{DR}}_{\text{GSR}}$ with varying BW and ES was assessed with an analysis of variance (ANOVA) corrected for multiple comparisons. As ANOVA revealed a significant difference the Spearman's rank correlation test was used to investigate any significant monotonic trend of the mean values of the measured parameters with increasing BW and ES.

## III. RESULTS

### A. Scanner‐A

**Figure 3:**
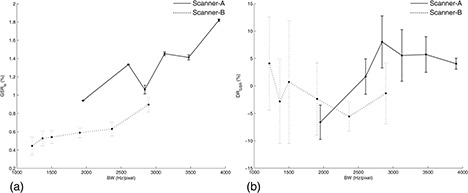
Scanner‐A (solid line): ${\text{GSR}}_{\text{ts}}$ (a) and ${\text{DR}}_{\text{GSR}}$ (b) as a function of BW at fixed ES=0.9ms. Scanner‐B (dotted line): ${\text{GSR}}_{\text{ts}}$ (a) and ${\text{DR}}_{\text{GSR}}$ (b) as a function of BW at fixed ES=0.9ms. Graphs report the mean and standard deviation of the three measurements performed for each BW value.

**Figure 4:**
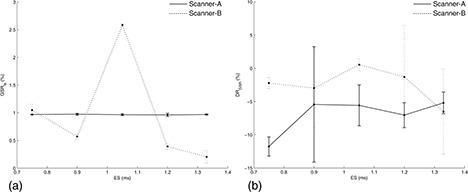
Scanner‐A (solid line): ${\text{GSR}}_{\text{ts}}$ (a) and ${\text{DR}}_{\text{GSR}}$ (b) as a function of ES at fixed BW=1953Hz/pixel. Scanner‐B (dotted line): ${\text{GSR}}_{\text{ts}}$ (a) and ${\text{DR}}_{\text{GSR}}$ (b) as a function of BW at fixed BW=1502Hz/pixel. Graphs report the mean and standard deviation of the three measurements performed for each ES value.

Analysis of variance revealed that ${\text{GSR}}_{\text{ts}}(p<0.000001)$ and ${\text{DR}}_{\text{GSR}}(p=0.008)$ significantly varied with BW. The mean ${\text{GSR}}_{\text{ts}}(r=0.886,\;p=0.019)$ significantly increased with increasing BW (Fig. 3(a)). The mean ${\text{DR}}_{\text{GSR}}$ increased with increasing BW (Fig. 3(b)), but the upward trend was not significantly monotonic (r=0.486,p>0.05). Both ${\text{GSR}}_{\text{ts}}(p>0.05)$ and ${\text{DR}}_{\text{GSR}}(p>0.05)$ did not significantly vary with ES (Fig. 4).

### B. Scanner‐B

Analysis of variance revealed that ${\text{GSR}}_{\text{ts}}(p=0.0004)$ significantly varied with BW, whereas ${\text{DR}}_{\text{GSR}}(p>0.05)$ did not (Fig. 3). The mean ${\text{GSR}}_{\text{ts}}(r=1,\;p<0.05)$ significantly increased with increasing BW (Fig. 3(a)). ${\text{GSR}}_{\text{ts}}(p<0.000001)$ significantly depended on ES, whereas ${\text{DR}}_{\text{GSR}}(p>0.05)$ did not (Fig. 4). The mean ${\text{GSR}}_{\text{ts}}$ decreased with increasing ES, but the downward trend was not significantly monotonic (r=‐0.705,p>0.05).

## IV. DISCUSSION

Although typical acquisition parameters (TR, TE, matrix, slice thickness, etc.) are generally employed in EPI‐fMRI sequences, various BW values are used as a function of gradients characteristics of the MR scanner system. Previous studies have investigated in vivo the dependence of BOLD activations on bandwidth. Fera et al.^(^ ^24^ ^)^ have suggested that the employment of low acquisition bandwidth can marginally increase the extent and significance of functional activations in motor task. Zou et al.^(^ ^25^ ^)^ have demonstrated for visual stimulus that larger activation size with lower average BOLD signal changes may be expected with EPI‐fMRI sequences having lower BW values for images that are not spatially smoothed, or for spatially smoothed images if strict statistical thresholds are used for activation map.

Echo spacing is a fundamental parameter of EPI acquisition sequences. In particular, the use of shorter ES allows the echo train length to be shortened with potential advantages in terms of reduced blurring and off‐resonance artifacts.^(^ ^30^ ^)^ However, EPI‐fMRI acquisitions at shorter ES should be efficiently corrected for eddy currents which may degrade image quality. We notice that, in EPI‐fMRI studies, the employed ES value is not usually reported.

Nyquist ghost can affect the overall quality and temporal stability of fMRI time series, which represent main prerequisites for successful studies of fMRI.^(^ ^39^ ^)^ Nyquist ghost can occur in EPI image reconstructions because of various factors such as gradient eddy currents, imperfect pulse sequence timing, magnetic field inhomogeneity and susceptibility effects. Thus, intensity and temporal stability of Nyquist ghost in EPI‐fMRI acquisitions can potentially vary with BW and ES. In particular, any temporal drift of Nyquist ghost is expected to depend on thermal stability of the MR scanner system during EPI‐fMRI acquisition.

Porter et al.^(^ ^40^ ^)^ have investigated the effect of residual Nyquist ghost in quantitative spin‐echo EPI‐diffusion imaging. They have shown that Nyquist ghost can produce severe artifacts such as regions with apparently low ADC (apparent diffusion coefficient) values, which simulate regions of reduced diffusion. Moreover, Jahng et al.^(^ ^41^ ^)^ have revealed at 4 T that diffusion anisotropic indexes are sensitive to BW of spin‐echo EPI‐diffusion acquisition sequence due to associated Nyquist ghost. Recently, Jahng and Schuff^(^ ^42^ ^)^ have studied at 4 T the influence of BW on quantitative gradient‐echo EPI‐perfusion measurements based on arterial spin labeling (ASL) technique. They have shown that Nyquist ghost can bias cerebral blood flow (CBF) quantification.

In a recently published article,^(^ ^43^ ^)^ we have studied the dependence of temporal stability and overall image quality of typical gradient echo EPI‐fMRI time series on readout bandwidth and echo spacing for two clinical 1.5 T MR scanner systems (scanner‐A, scanner‐B). The temporal stability of EPI‐fMRI sequence has been assessed measuring the signal‐to‐fluctuations noise ratio (SFNR) and signal drift (DR), while the overall image quality has been assessed evaluating the signal‐to‐noise ratio (${\text{SNR}}_{\text{ts}}$) and non‐uniformity (${\text{NU}}_{\text{ts}}$) of the time series acquisition. For both scanners no significant effect of BW and ES on signal drift has been revealed. On the other hand, SFNR, ${\text{SNR}}_{\text{ts}}$ and ${\text{NU}}_{\text{ts}}$ can significantly vary with BW and ES. In the present study, we specifically investigated the effect of both readout bandwidth and echo spacing on Nyquist ghost of gradient echo EPI‐fMRI acquisitions. For scanner‐A (93% of variation) and scanner‐B (102% of variation), ${\text{GSR}}_{\text{ts}}$ significantly increased with increasing BW. Since the strength of readout gradient ($G_{r}$) linearly increases with increasing BW (Eq. 1), eddy currents effect is more pronounced at higher readout bandwidth values. Moreover, the frequency response characteristic of the signal receiver system has been described as a contributing factor to the formation of Nyquist ghost in EPI acquisitions.^(^ ^44^ ^)^ Thus, the ${\text{GSR}}_{\text{ts}}$ results can be explained also in terms of the variation of the frequency response characteristic within the frequency range corresponding to readout bandwidth. Indeed, at reduced BW values, the variation of the frequency response characteristic is smaller because the image frequencies are limited within a smaller range. The revealed variation of ${\text{GSR}}_{\text{ts}}$ as a function of BW is in agreement with the water phantom data reported by Delakis et al.,^(^ ^44^ ^)^ which were obtained using TR (1300 ms) and TE (180 ms) values not typically employed in clinical fMRI acquisitions. Two previous studies at 4 T have reported data concerning the effect of BW on intensity of Nyquist ghost in spin‐echo EPI‐diffusion^(^ ^41^ ^)^ and gradient‐echo EPI‐perfusion sequences.^(^ ^42^ ^)^ However, in these studies BW values were not varied independently of ES values, and a comparison with our results cannot be performed.

${\text{GSR}}_{\text{ts}}$ values of scanner‐A did not significantly depended on ES. On the other hand, ${\text{GSR}}_{\text{ts}}$ values of scanner‐B significantly varied with ES. Ghost‐to‐signal ratio values of scanner‐B showed a downward trend (81% of variation), with increasing ES from 0.75 ms to 1.33 ms. This seems to suggest a suboptimal EPI correction for eddy currents, which is likely to increase intensity of Nyquist ghost at shorter ES. We observed a ${\text{GSR}}_{\text{ts}}$ spike point at ES=1.05ms, which may be due to a miscalibration of pulse sequence timing. Nonetheless, it cannot be ignored that an increase of Nyquist ghost due to magnetic field fluctuations might be caused by greatly increased mechanical vibrations of readout gradient coil at ES=1.05ms. Indeed, previous studies^(^ ^45^ ^,^ ^46^ ^)^ have shown that the oscillating magnetic field gradient can produce considerable mechanical vibrations as the readout frequency (1/ES) coincides with one of the resonance modes of the gradient coil, which depend on the coil length and elastic properties of the materials in the assembly.

**Figure 5:**
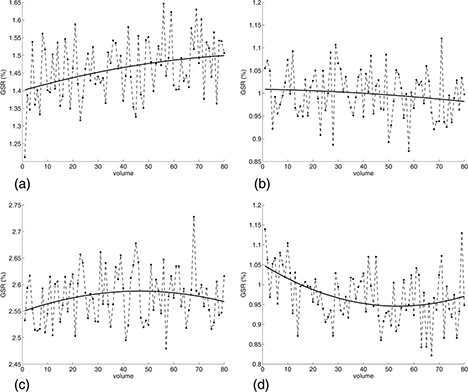
Temporal variation of ghost‐to‐signal ratio values during EPI‐fMRI acquisitions superimposed with the second order polynomial trend fitted to time series data points: (a) scanner‐A, BW=3125Hz/pixel,ES=0.9ms; (b) scanner‐B, BW=1502Hz/pixel,ES=0.75ms; (c) scanner‐B, BW=1502Hz/pixel,ES=1.05ms; (d) scanner‐A, BW=1953Hz/pixel,ES=0.75ms.

No significant effect of ES on drift of ghost‐to‐signal ratio was revealed for either scanner. ${\text{DR}}_{\text{GSR}}$ values of scanner‐B did not significantly vary with BW, whereas ${\text{DR}}_{\text{GSR}}$ values of scanner‐A significantly depended on BW showing an upward trend from negative to positive values with increasing readout bandwidth. ${\text{DR}}_{\text{GSR}}$ values of scanner‐A sharply increased for BW of less than 3000 Hz/pixel, while they remained almost unchanged for BW greater than 3000Hz/pixel. The drift of ghost‐to‐signal ratio depends on warming gradients effect in high‐duty cycle acquisition sequences and thermal stability of the MR scanner system during EPI‐fMRI acquisition. As BW increases, oscillating readout gradients with higher strength are employed, resulting in a potential greater thermal stress of gradients hardware. For both scanner‐A and scanner‐B, we revealed monotonic (Fig. 5(a)–5(b)) and non‐monotonic (Fig. 5(c)–5(d)) polynomial trend fitted to ghost‐to‐signal ratio values of EPI‐fMRI time series.

For each MR scanner system, the basic effect of BW and ES on Nyquist ghost of EPI‐fMRI acquisition sequences can be characterized by means of phantom measurements of ${\text{GSR}}_{\text{ts}}$ and ${\text{DR}}_{\text{GSR}}$. We note that these measurements should be considered with caution in order to optimize EPI‐fMRI sequences for examinations in clinical studies. Indeed, phantom acquisitions do not take into account effects of magnetic field fluctuations due to subject motion during scan, and they are not affected by physiological noise. In addition, a homogenous phantom cannot properly simulate warping effects in subjects' acquisitions, which are mostly pronounced at boundaries of regions with high contrast and produce artifacts such as those present along cortical sulci in EPI‐fMRI images. Nonetheless, differences in performances of MR scanner system are potential source of variability for fMRI activation.^(^ ^39^ ^,^ ^47^ ^)^ Therefore, in order to improve the reliability of group comparison and longitudinal studies of fMRI, the MR scanner system stability as regards Nyquist ghost should be monitored by means of measurements of ${\text{GSR}}_{\text{ts}}$ and ${\text{DR}}_{\text{GSR}}$.

## V. CONCLUSIONS

The intensity of Nyquist ghost (${\text{GSR}}_{\text{ts}}$) and its temporal variation (${\text{DR}}_{\text{GSR}}$) in EPI‐fMRI time series can significantly vary with readout bandwidth and echo spacing. The specific pattern of variation may depend on each single MR scanner system in terms of gradient characteristics, EPI sequence calibrations (eddy current, shimming, etc.) and functional design of radiofrequency coil. Our results indicate that the employment of low BW values seems to reduce the intensity and temporal variation of Nyquist ghost of EPI‐fMRI time series. On the other hand, the use of minimum ES value would be not entirely advantageous when the MR scanner is characterized by gradients with low performances and suboptimal EPI sequence calibrations. Nyquist ghost may greatly affect fMRI time series at specific ES values owing to a potential resonant effect.

## ACKNOWLEDGEMENTS

The authors would like to thank Piero Ghedin (GE Healthcare) for the expert technical assistance, and Ilaria Pesaresi (University of Pisa) for kind support during data acquisition.
